# Thermal patterns in stingless bee colonies

**DOI:** 10.1007/s00114-026-02083-6

**Published:** 2026-03-03

**Authors:** Charles Fernando dos Santos, Kedar Devkota, Betina Blochtein, Eduardo A. B. Almeida

**Affiliations:** 1 Laboratório de Abelhas e Polinização, Departamento de Fitossanidade, Faculdade de Agronomia, Universidade Federal do Rio Grande do Sul, Porto Alegre, Brazil; 2 Faculty of Agriculture, Agricultural and Forestry University, Chitwan, Nepal; 3 Mais Abelhas Consultoria Ambiental Co, Porto Alegre, Brazil; 4 Laboratório de Biologia Comparada e Abelhas, Departamento de Biologia, Faculdade de Filosofia, Ciências e Letras de Ribeirão Preto, Universidade de São Paulo, Ribeirão Preto, São Paulo, 14040-901 Brazil

**Keywords:** Brood thermoregulation, Meliponiculture, Nest architecture, Phylogenetic Comparative Methods

## Abstract

Understanding the thermal dynamics of stingless bee colonies is essential for developing effective monitoring systems and promoting sustainable meliponiculture. In this study, we compiled and analyzed brood, nest periphery, and ambient temperature data from 36 stingless bee species reported across several published studies. The data were systematized into graphical visualizations, phylogenetic comparative methods, Generalized Least Squares both for heteroscedasticity or autocorrelation in the errors, coefficients of variation (CV), and calculated temperature deltas (ΔT), i.e. the differences between distinct nest zones, to evaluate thermal regulation and summarize temperature patterns across a broad diversity of stingless bees. Our findings show that species with defined brood combs and cerumen envelopes tend to maintain significantly higher and more stable brood temperatures, averaging close to 30 °C, compared to species lacking these structures. Nest temperatures in stingless bees showed a weak but significant phylogenetic signal, indicating partial evolutionary structuring of thermal traits. Yet, the presence of an involucrum was a strong predictor of internal colony temperature, with species constructing this structure maintaining nests approximately 4.8 °C warmer than those lacking it. These results suggest that involucrum construction is a key adaptive trait that largely overrides phylogenetic constraints on thermal regulation. Thermal stability was highest in brood areas (CV = 11.7%) and progressively decreased toward the outer nest and ambient environment (CV = 35.3%), indicating strong internal buffering mechanisms. These findings identify the involucrum as a key adaptation driving elevated and stable brood temperatures in stingless bees, largely independent of phylogenetic constraints, with important implications for thermal biology, monitoring, and sustainable meliponiculture.

**Supplementary Information:**

The online version contains supplementary material available at 10.1007/s00114-026-02083-6.

## Introduction

Bees are essential pollinators in tropical ecosystems and play an prominent role in ecological restoration, agroecology, and urban biodiversity efforts (Potts et al. 2016; Lichtenberg et al. 2017). Approximately 40% of insect pollinators, such as bees and butterflies, and 16% of vertebrate pollinators, such as birds and bats, are at risk of extinction due to habitat loss, pesticide use, pests and pathogens, pollution, and climate change (IPBES 2016). As climate change intensifies (Giannini et al. 2017; Jaffé et al. 2019), ensuring the survival of bee colonies may become increasingly dependent on targeted conservation strategies. In this context, understanding the thermal tolerance of native social bee species, particularly those widely managed for pollination, is essential. Knowing the average temperature within their nests may offer valuable insights into how these bees regulate and adapt to thermal fluctuations (Dantas 2016; Lima et al. 2021; Nacko et al. 2023). Such knowledge is important for developing accurate species distribution models that incorporate thermal variation across populations (dos Santos et al. 2015) and serve as a baseline for real-time, automated, and long-term monitoring and conservation under changing climate conditions (Lima et al. 2021).

Among social bees, stingless bees (Hymenoptera: Apidae: Meliponini) are the most diverse and species-rich group (Engel et al. 2023; Lepeco et al. 2024). Understanding the internal conditions of their nests is vital for supporting their health and sustainability in managed environments. Among various environmental parameters, temperature is a critical factor affecting all aspects of colony functioning, i.e. brood development, foraging behavior and long-term survival (Velthuis et al. 1999; Jones and Oldroyd 2006; Vollet-Neto et al. 2014; Roldão-Sbordoni et al. 2024).

In contrast to the extensively studied honey bee (*Apis mellifera*), stingless bees generally exhibit a limited capacity for active thermoregulation, such as fanning or evaporative cooling (Velthuis et al. 1999; Jones and Oldroyd 2006; Vollet-Neto et al. 2014; Dantas 2016; Roldão-Sbordoni et al. 2024). Although some stingless bee species may display rudimentary forms of active thermoregulation, they are generally less developed and less effective than those observed in honey bees (Velthuis et al. 1999; Jones and Oldroyd 2006; Vollet-Neto et al. 2014; Dantas 2016; Roldão-Sbordoni et al. 2024). Instead, stingless bees rely on passive thermoregulatory strategies such as nest architecture, material composition, and worker behavior, including the construction of cerumen envelopes around the brood and the spatial distribution of individuals within the nest to maintain favorable microclimatic conditions (Fletcher and Crewe 1981; Engels et al. 1995; Velthuis et al. 1999; Jones and Oldroyd 2006; Vollet-Neto et al. 2014; Dantas 2016; Roldão-Sbordoni et al. 2019).

The brood area, where immature bees are raised, is particularly sensitive to thermal variations. Temperature fluctuations in this brood area can have significant consequences on larval development, adult morphology, and colony productivity (Fletcher and Crewe 1981; Roubik et al. 1983; Dantas 2014; Roldão-Sbordoni et al. 2019). Suboptimal or extreme temperatures have been associated with developmental delays, physiological deformities, and increased mortality rates (Fletcher and Crewe 1981; Roubik et al. 1983; Dantas 2014; Becker et al. 2018; Roldão-Sbordoni et al. 2019). In addition, temperatures exceeding 35 °C can melt cerumen structures, while suboptimal temperatures below 20 °C may trigger reproductive diapause or developmental arrest in brood (Fletcher and Crewe 1981; Roubik et al. 1983; Dantas 2014; Becker et al. 2018; Roldão-Sbordoni et al. 2019). Such temperature stress can also affect behavior, for example, by inducing precocious foraging, and may further compromise the immune system. Experimental evidence shows that workers exposed to heat stress begin foraging significantly earlier but live shorter lives, while males experience a marked reduction in immune response, which together may have cascading impacts on colony fitness (Quezada-Euán et al. 2024).

Internal thermoregulation may be affected by species-specific traits, such as body size, colony population, and behavioral plasticity of stingless bees. These intrinsic factors influence the colony’s ability to maintain thermal stability in the face of external temperature variations (Gonzalez et al. 2022). For instance, it is known that a stingless bee species (*Melipona scutellaris*) exhibits ‘warming nurses’, i.e., workers that increase thoracic temperature near the brood to support its development (Roldão-Sbordoni et al. 2019).

In order to support advancements in stingless beekeeping, we conducted an extensive literature review on nest temperatures across a wide range of Meliponini species. Our aim was to compile and synthesize internal temperature data, especially in the brood area, nest periphery, and when available, the external environmental conditions surrounding the nests of the various stingless bee species. We present a systematized temperature record from the literature within an exploratory framework to guide future research and practical applications in stingless bee management. When data were available, we conducted in addition phylogenetic comparative methods (see below).

## Materials and methods

To conduct this surveying, we employed search terms in both Portuguese and English related to “stingless bees,” “temperature,” and “thermoregulation.” The literature search was performed across multiple academic databases, including the Web of Science, SciELO (Scientific Electronic Library Online), and Google Scholar. No temporal restrictions were applied, allowing the inclusion of studies published at any point in time. In some instances, the only sources of relevant data were unpublished academic works, such as master’s dissertations or doctoral theses. These were also included in our review, provided they met our inclusion criteria.

The primary condition for inclusion in the study was that it must have assessed, within the same investigation, the average temperature and thermal variability across three key areas: the brood area (bee combs), the nest periphery, and the external environmental conditions surrounding the nests of stingless bees. This criterion ensured consistency and comparability of thermal profiles across different species and nest structures. Additionally, whenever available, we extracted supplementary information on nest architecture, particularly the structural configuration of the brood comb. The term brood comb is used here in a broad sense, encompassing both organized arrangements layered horizontal formations and those more amorphous clusters of brood cells, which typically lack a covering involucrum and are not arranged in well-defined sheets. The presence or absence of a cerumen envelope surrounding the brood was not systematically recorded, as it often correlates with brood comb structure. With few exceptions, cerumen envelopes are typically present in species that construct well-defined combs and absent in those that form loosely arranged brood clusters.

All analyses and data visualizations were performed using the R statistical computing environment (Ihaka and Gentleman 1996; R Core Team 2024). We assessed thermal variability within the nests by calculating the coefficient of variation (CV) for each thermal zone: brood area, nest periphery, and external surrounding environment. This allowed us to evaluate the relative stability of temperatures across these zones. Additionally, we computed temperature deltas (Δ) to quantify the differences between the brood area and both the nest periphery (Δbrood–periphery) and the external surrounding environment (Δbrood–ambient). These measures provided insight into the degree of thermal buffering offered by nest architecture and materials.

### Statistical analysis

#### Phylogenetic comparative analyses

For the comparative analysis of the 36 species of Meliponini considered in this investigation, we used a phylogenetic framework based on the findings of research on the systematics of stingless bees (Ramírez et al. 2010; Rasmussen and Cameron 2010; Lepeco et al. 2024). Most branch lengths were estimated proportionally to time, except for the relationships among some species of *Friseomelitta*, *Melipona (Michmelia)*, *Scaptotrigona*, and *Tetragonisca*. In these cases, closely related species were clustered within their respective clades, and a divergence time close to zero was assigned, resulting in soft polytomies that reflected our uncertainty about their specific relationships (Garland and Díaz-Uriarte 1999). Subsequently, we added additional duplicated tips from most species to reach the 113-terminal tree required for the subsequent analyses (Supplementary Material), where multiple tips of the same species were placed as unresolved zero-branch length clusters. The full 36-species chronogram was pruned to include only the 24 species needed for the analyses (Supplementary Material) in which involucrum is known to occur or not.

Phylogenetic comparative analyses were conducted at the species level to account for the non-independence of trait values arising from shared evolutionary history. Analyses were performed both considering all species collectively and by explicitly comparing species that construct an involucrum enclosing the brood area with those that do not, in order to evaluate potential differences in thermal regulation strategies. First, we quantified the strength of phylogenetic signal in mean nest temperature across the stingless bee phylogeny using Pagel’s λ, thereby assessing the extent to which interspecific variation in thermal traits is structured by phylogenetic relatedness. Phylogenetic signal was estimated by maximum likelihood and significance was assessed via randomization using 9,999 simulations, as implemented in `phytools::phylosig` (Revell 2012). This step evaluated whether temperature values were more similar among closely related species than expected under phylogenetic independence.

Yet, to test whether mean temperature differed between groups defined by the presence/absence of an involucrum, we fit a phylogenetic generalized least squares model (PGLS) using the `caper` package (Orme et al. 2013). We assembled a comparative dataset with `caper::comparative.data`. We then modeled temperature as a function of involucrum status using `caper::pgls`, estimating Pagel’s λ by maximum likelihood (λ = `ML`). For visualization, we mapped trait values onto the phylogeny by constructing a `phylo4d` object (tree plus associated temperature data) using `phylobase::phylo4d` (Bolker et al. 2024). We subsequently generated phylogeny-aligned barplots with `barplot.phylo4d`, displaying temperature values at the tips in the same order as the phylogeny to facilitate qualitative assessment of phylogenetic structure and group-level contrasts.

#### Temperature deltas (Δ) among brood, periphery, and environment

To investigate temperature differences within nests, brood temperature was analyzed in relation to periphery and environmental temperatures. We tested homoscedastic and heteroscedastic models with variance structures linked to predictors or fitted values, selecting the best model by AIC (Akaike information criterion). To compare mean values directly, the dataset was reformatted to long structure and analyzed with a GLS model implemented in the `nlme` package (Pinheiro et al. 2020) including compound symmetry correlation and heterogeneous residual variances by location. Post-hoc comparisons were obtained with the `emmeans` package (Lenth 2019), and p-values adjusted by the false discovery rate (FDR) method to account for multiple comparisons between brood, periphery, and environment. The R scripts employed to perform these GLS analyses are provided in the Supplementary Material.

## Results

**Fig. 1:**
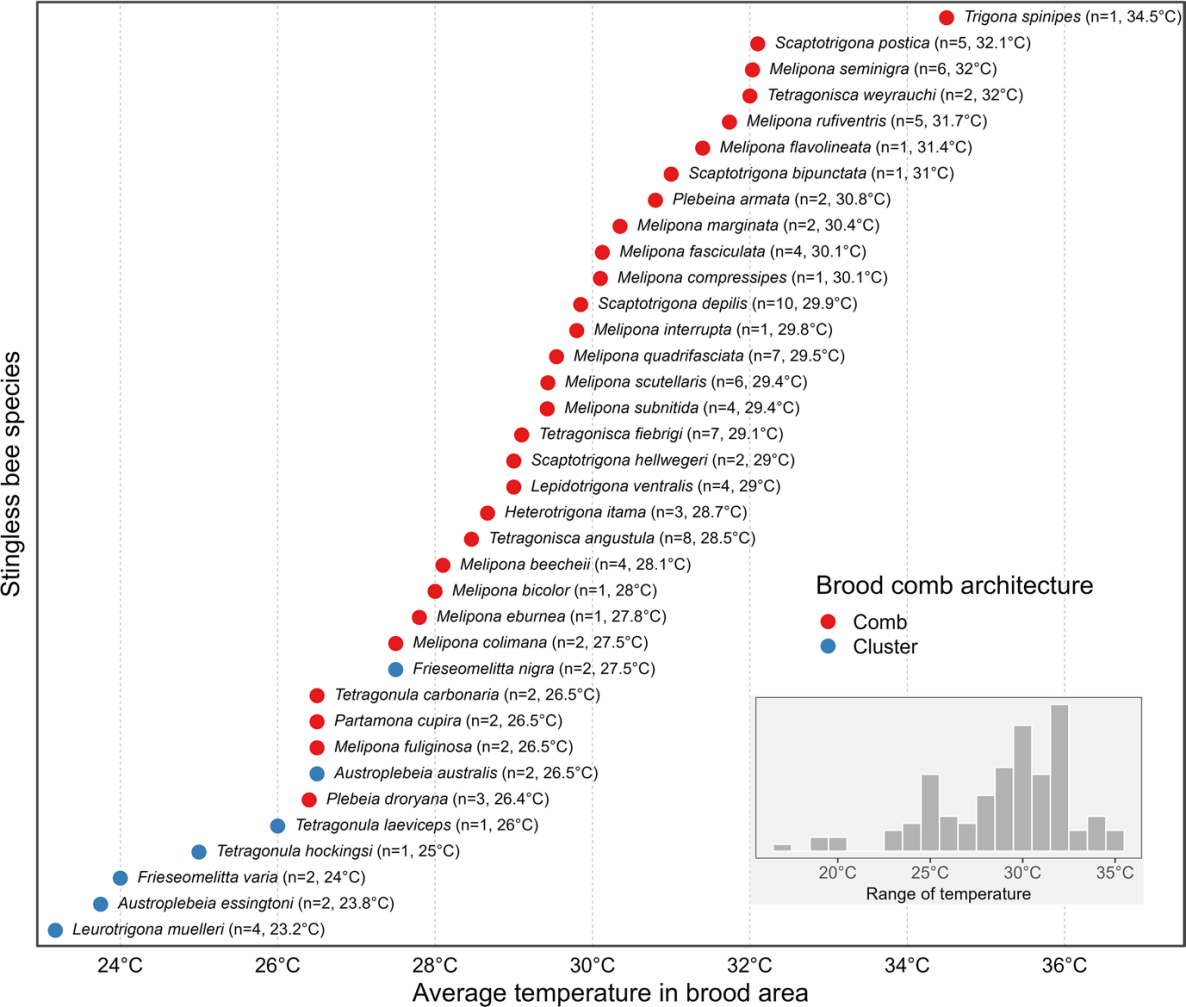
Average temperature in the brood area of stingless bee species, sorted by increasing mean temperature. Each dot represents the mean internal temperature of the brood area for a given species. Red points indicate species with brood combs, while blue points represent species with clusters. Species names are followed by the number of studies sampled and the average temperature in degrees Celsius. The inset histogram in the lower right corner shows the overall distribution of average brood temperatures across all species in the dataset

Overall, we recorded 36 distinct stingless bee species across 113 individual observations (see Supplementary Material for literature). Our main findings indicate that species that construct brood combs tend to maintain higher internal brood temperatures, typically above 28 °C and frequently reaching or exceeding 30 °C (Fig. 1). In contrast, species that exhibit amorphous brood cluster architecture generally exhibit lower brood area temperatures, predominantly ranging between 23 °C and 27 °C (Fig. 1).

Among the data availability, the findings show that *Leurotrigona muelleri* exhibits the lowest recorded brood temperature (23.2 °C), while *Trigona spinipes* shows the highest (34.5 °C). Interestingly, multiple species of the genus *Melipona* cluster around similar to each other internal temperature ranges, typically between 28 °C and 31 °C (Fig. 1). This pattern may reflect phylogenetic constraints or conserved evolutionary strategies underlying brood thermal regulation in this genus.

Additionally, while some species, such as *Scaptotrigona depilis* and *Melipona compressipes*, are supported by several observations, others are represented one or two records (Fig. 1). Consequently, caution is warranted when interpreting patterns for species with limited sample representation, as these values may not adequately captured their intraspecific variability. More sampling and replication will be essential to strengthen the generality of the observed trends.

**Fig. 2:**
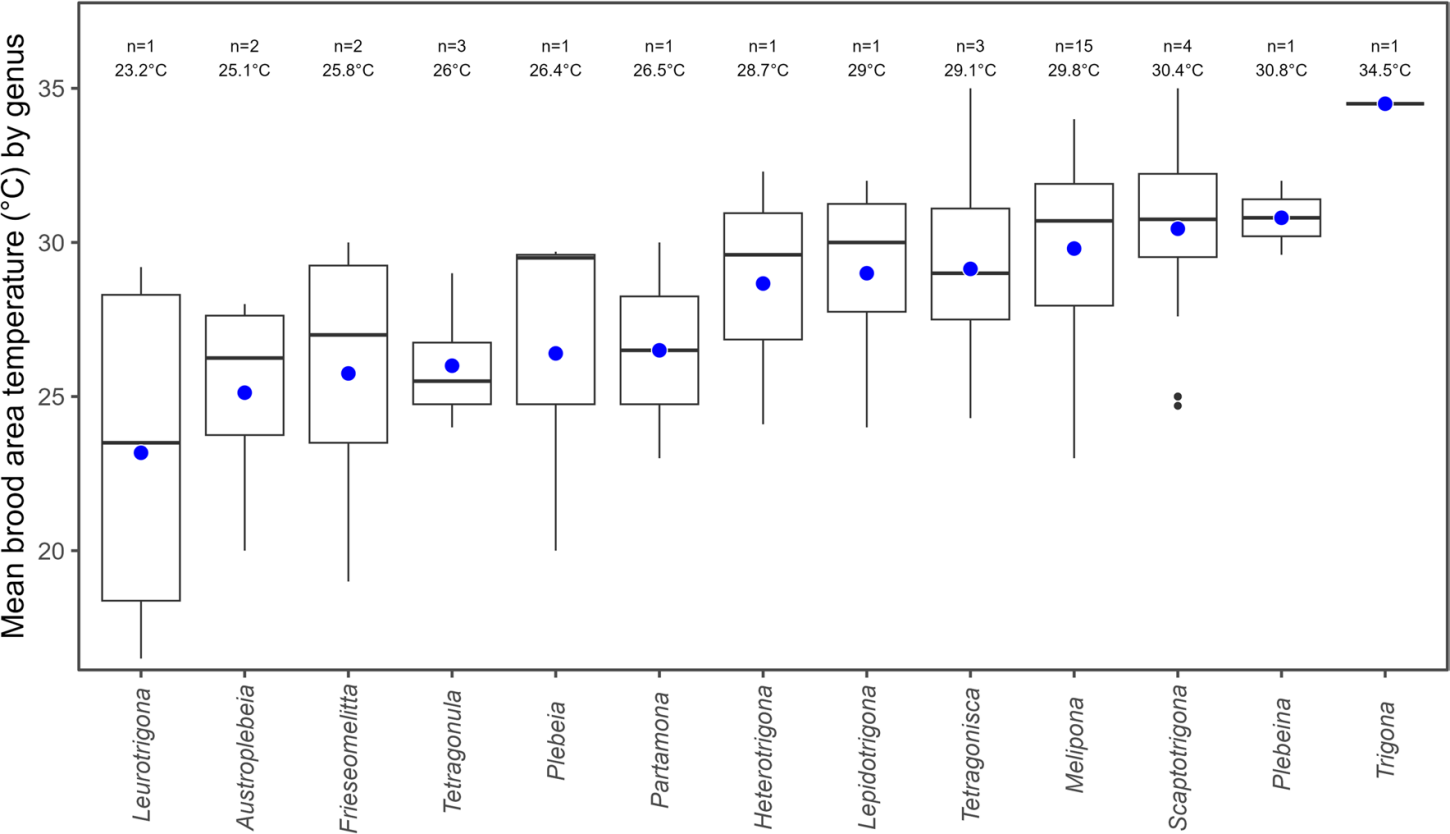
Mean temperature in the brood area of stingless bee colonies grouped by genus. Boxplots show the distribution of brood-area temperatures for each genus (thick horizontal line = median; box = 25th–75th percentiles; whiskers = ± 1.5× IQR; black dots = outliers). Blue circles mark the genus mean. The annotation above each box reports the number of species contributing to that genus (n) [a few species appear as singletons in our dataset, although they occur in diverse contexts] and the corresponding mean (°C). Genera are ordered left-to-right by increasing mean temperature. Temperatures were compiled from published records (see Supplementary Material)

From a genus-level perspective, we found taxa from South America, Central America, Mexico, Africa, Asia (Southeast Asia), and Australia. The thermal results reveal substantial variation in brood area temperature among stingless bee genera. *Trigona* exhibits the highest recorded mean brood temperature (34.5 °C). Other genera, including *Tetragonisca*, *Scaptotrigona*, and *Melipona*, also maintain relatively high brood temperatures, typically exceeding 29 °C. In contrast, genera such as *Leurotrigona*, *Austroplebeia*, and *Frieseomelitta* show consistently lower brood temperatures, often below 26 °C (Fig. 2).

Notably, *Melipona* demonstrates both higher mean temperatures and a narrower interquartile range, suggesting greater stability and consistency in brood thermoregulation across species. In contrast, *Lepidotrigona* exhibits broader temperature variability. It is also important to interpret results cautiously: for genera with limited data (e.g., *Trigona*), small sample sizes may not fully capture intra-generic variation, and apparent trends may be influenced by ecological and climatic adaptations rather than sampling alone. (Fig. 2).

**Fig. 3:**
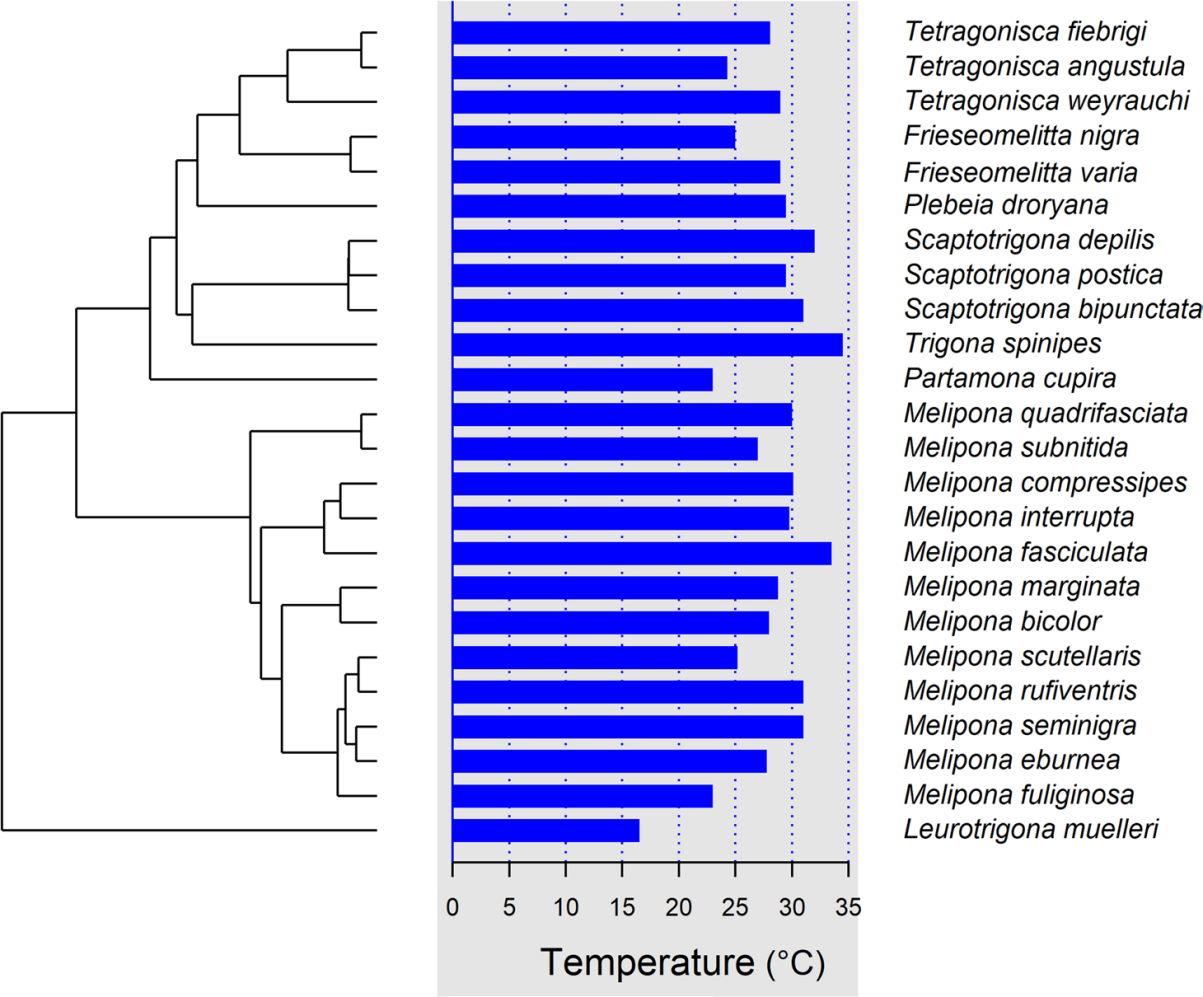
Phylogenetic distribution of mean internal colony temperature (brood area) in stingless bees. The phylogenetic tree (left) represents evolutionary relationships among 24  Neotropical stingless bee species, while horizontal bars (right) indicate species-level mean nest temperature (°C). Bars are aligned with corresponding terminal taxa in the phylogeny, allowing direct visualization of thermal variation in relation to evolutionary history

Pagel’s λ indicated a weak but statistically significant phylogenetic signal (λ = 0.22; LR test against λ = 0: χ² = 5.66, *P* = 0.017) – Fig. 3, suggesting that thermal traits are somehow partially structured by phylogenetic relatedness.

**Fig. 4:**
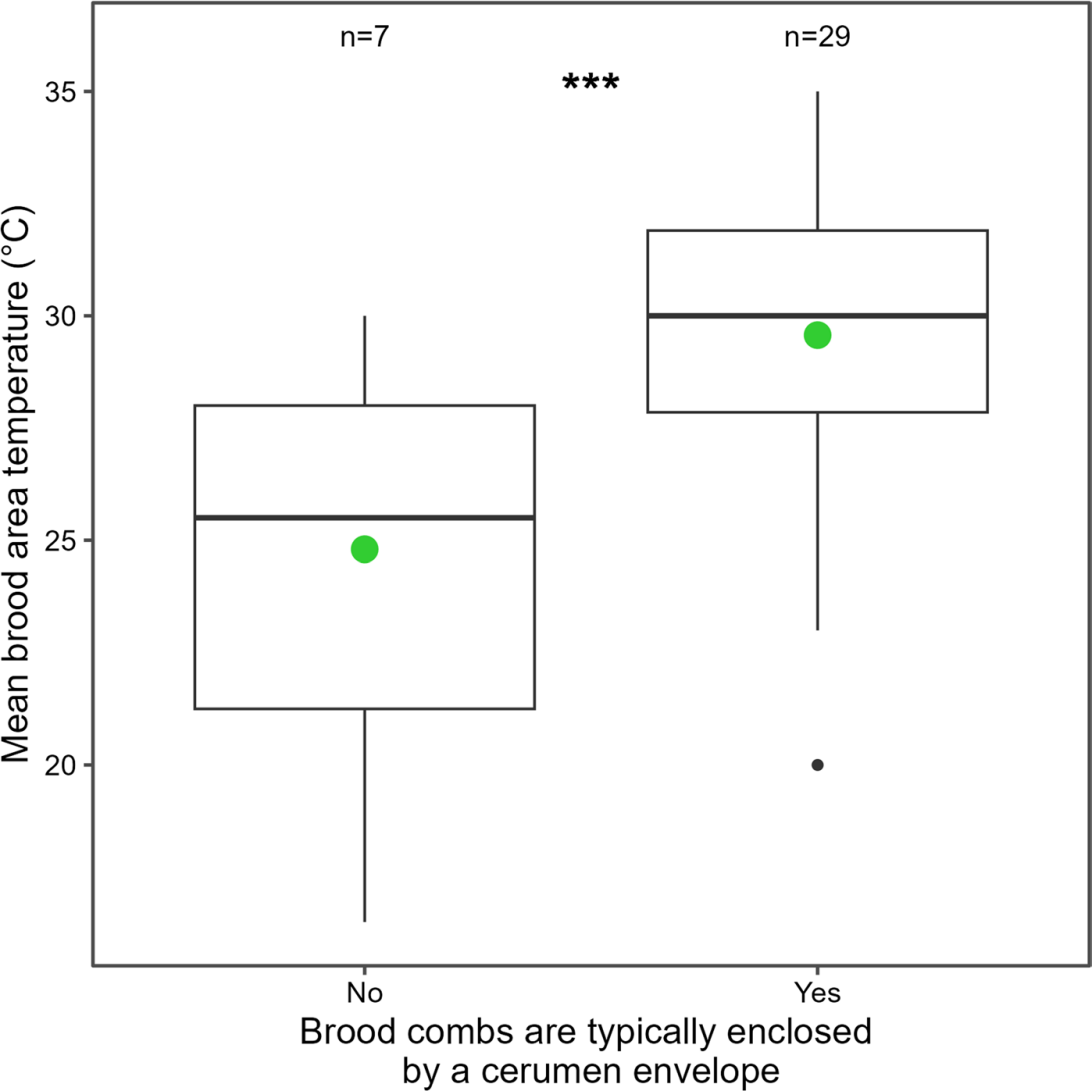
Comparison of mean brood area temperatures between stingless bee species. Boxplots summarize temperatures for species without (`No´) and with (`Yes´) a cerumen envelope around the brood combs (No = cluster lacking a protective layer; Yes = combs enclosed by cerumen). The thick horizontal line is the median; boxes show the interquartile range (IQR); whiskers extend to 1.5×IQR; isolated points are outliers. Green circles mark the group mean. Values above boxes indicate the number of species contributing to each group (not the number of records; See Supplementary Material). Asterisks denote statistical significance at *p* < 0.001

However, phylogenetic generalized least squares analysis revealed a strong association between the presence of an involucrum and mean internal colony temperature. Species that construct an involucrum enclosing the brood exhibited significantly higher nest temperatures than species lacking this structure (β = 4.85 °C ± 1.29 SE; t = 3.76; *P* = 0.001), Fig. 4. On average, colonies without an involucrum maintained temperatures around 24.9 °C, whereas the presence of an involucrum was associated with an increase of nearly 5 °C in internal nest temperature. Notably, the maximum-likelihood estimate of Pagel’s λ was zero, indicating that after accounting for involucrum presence, residual variation in nest temperature showed no detectable phylogenetic structure. This suggests that the effect of involucrum construction on thermal regulation is largely independent of shared evolutionary history and represents a convergent functional strategy across stingless bee lineages.

The coefficients of variation calculated for the three temperature zones (brood, nest periphery, and surrounding environment) provided valuable insights into the stability of thermal conditions within stingless bee nests. The brood area exhibits the lowest coefficient of variation (CV = 11.7%), indicating relatively stable temperatures in this central part of the nest. In contrast, the net periphery showed a higher CV (23.3%), reflecting greater temperature fluctuations. This region may be less protected or less actively regulated, serving as a buffer zone between the well-regulated brood area and the external environment. As expected, the external environment exhibits the highest variability (CV = 35.3%), which may be due to its direct exposure to natural weather conditions.

Overall, the brood area not only exhibited the lowest coefficient of variation but also maintained a relatively high and stable mean temperature of 30.4 °C ± 3.57 standard deviation (SD). In comparison, the nest periphery exhibited a lower mean temperature of 25.6 °C ± 5.98 SD, while the external surrounding environment showed the lowest mean temperature (23.5 °C ± 8.32 SD), suggesting that temperature variability increases progressively from the brood center outward to the external surrounding environment. Therefore, the GLS model revealed significant temperature differences among locations, with the brood warmer than the periphery and environment, and the periphery also warmer than the environment (all *p* < 0.05).

Our analysis of temperature differentials within the colonies revealed a marked degree of thermal buffering in stingless bee nests. On average, the brood area was 4.8 °C ± 4.1 SD warmer than the nest periphery (Δbrood–periphery), demonstrating that colonies actively maintain a more favorable microclimate for brood development compared to the surrounding internal nest space. Compared with the external environment, the brood area exhibited an even stronger buffering capacity, maintaining an average temperature 6.8 °C ± 6.1 SD higher than the environment (Δbrood–environment). Additionally, the nest periphery remained 2 °C ± 3.9 SD warmer than the external environment (Δperiphery–environment), indicating that even the outer regions of the colony provide some degree of thermal protection.

## Discussion

Our findings contribute to a growing body of evidence highlighting the complexity and effectiveness of thermoregulatory strategies among stingless bees. By demonstrating consistent thermal gradients across species and nest zones, and by quantifying variation with statistical rigor, this study provides the baseline on temperature dynamics within stingless bee colonies and their surrounding environment. The strong association between brood comb architecture, cerumen envelope presence, and thermal stability highlights the functional significance of nest structures in regulating microclimatic conditions.

In addition, interspecific variation in internal colony temperature was only partially structured by phylogenetic relatedness, with closely related species tending to exhibit somewhat similar thermal conditions, but with substantial divergence among lineages. The low magnitude of λ suggests limited evolutionary conservatism in thermal regulation, consistent with strong influences of ecological context, nesting architecture, and species-specific behavioral strategies. Nevertheless, the significant departure from phylogenetic independence demonstrates that evolutionary history still contributes to shaping thermal traits in stingless bee colonies and should be accounted for in comparative analyses.

The observed interspecific variation in brood comb temperature across studies likely reflects differences in nest architecture, colony size, microhabitat conditions, and phylogenetic constraints. Notably, the results indicate that a cerumen envelope enhances thermal stability in the brood area. In contrast, cluster species exhibit lower temperatures, likely reflecting the absence of this protective cerumen layer, which may reduce their capacity to buffer against thermal fluctuations. This structure appears to function as an insulating barrier, buffering the brood from external temperature fluctuations (Roubik et al. 1983; Engels et al. 1995; Velthuis et al. 1999; Roldão-Sbordoni et al. 2024). The coefficient of variation further supports the role of this structure in thermal buffering. For example, the brood area exhibited the lowest variability (CV = 11.7%), suggesting effective thermal homeostasis, whereas temperatures outside the brood area but within the nest showed greater variability (CV = 23.3%). External environments were even more variable (CV = 35.3%). These patterns are consistent with findings from the surveyed literature, which indicate that internal nest regions maintain distinct and regulated microclimates (Loli 2008; Sung and Hozumi 2008; Becker et al. 2018; Caldas et al. 2024).

Although passive mechanisms like physiological or behavioral mechanisms are undoubtedly important for nest thermoregulation, numerous studies have consistently demonstrated that the central brood area of stingless bee nests is typically warmer than peripheral nest regions (Fletcher and Crewe 1981; Engels et al. 1995; Proni and Hebling 1996; Moo-Valle et al. 2000; Torres et al. 2007; Halcroft et al. 2013). This thermal gradient is also attributed to heat generated by the metabolic activity of brood development, namely larvae and pupae, as well as the clustering of adult workers in or near the brood region (Roubik et al. 1983; Engels et al. 1995; Roldão-Sbordoni et al. 2019). Nevertheless, the specific contribution of immature bees to the colony’s overall thermal dynamics remains underexplored. Hence, stingless bees appear to adopt a predominantly passive thermoregulatory strategy, relying less on active physiological control and more on structural features and microclimatic buffering to maintain brood area temperature (Jones and Oldroyd 2006; Roubik and Peralta 1983).

Unlike honey bees, which use elaborate and complex fanning and evaporative cooling (Engels et al. 1995; Stabentheiner et al. 2021), stingless bees rely on architecture and clustering behavior (Nieh and Sánchez 2005; Macías-Macías et al. 2011; Roldão-Sbordoni et al. 2019). We found that brood area temperatures were consistently higher and more stable than those in the nest periphery and external surrounding environment. This is evidenced by the progressive increase in the coefficient of variation, indicating that while nest temperatures fluctuate in response to external conditions, the brood area maintains relative thermal stability due to internal buffering mechanisms.

Temperature extremes, both high and low, can negatively affect colony function (dos Santos et al. 2014; Ferreira 2014; Vollet-Neto et al. 2014; Dantas 2016; Becker et al. 2018; Nacko et al. 2023; Robinson and Baudier 2024). Our results extend this knowledge by showing that most likely species with a cerumen envelope maintain narrower temperature ranges, suggesting a natural buffering capacity that may mitigate larval deformities within the brood area and queen oviposition decline under extreme heat, for example. Conversely, cluster species without this protection show greater variability, highlighting their potential vulnerability and the need for targeted management strategies. Therefore, hive materials and placement should be carefully considered, as they play critical roles in buffering colonies against climatic extremes.

Finally, factors such as hive materials, insulation capacity, placement, and species-specific requirements should be incorporated into future guidelines to ensure brood viability and overall colony performance. Furthermore, non-invasive monitoring using digital sensor systems must also account for other internal and external drivers of nest temperature, including colony population size, prevailing climatic conditions, hive construction materials, and the specific microenvironment in which hives are installed.

As a final recommendation, we suggest digital sensors, preferably over or within the brood area, as temperature measurements at the nest periphery tend to be highly variable and do not accurately reflect the thermal conditions required for brood development and survival. Moreover, adult bees often cluster near the brood during colder conditions or relocate and even remove the cerumen envelope when temperatures rise excessively. Ultimately, this data synthesis and exploratory analyses will support a new generation of stingless beekeepers, enabling more assertive, rapid, and precise colony management practices. These technological advancements can enhance colony survival and contribute to greater productivity of apicultural products and agricultural crop yields, as well as to pollination services.

## Supplementary Information

Below is the link to the electronic supplementary material.

Supplementary Material 1 (PDF 772 KB)

## Data Availability

Data will be available as a supplemental material.
