# Supplementary material for: Thermal patterns in stingless bee colonies
Source: Naturwissenschaften. 2026 Mar 3;113(2):29. doi: 10.1007/s00114-026-02083-6 (PMC12957120; doi:10.1007/s00114-026-02083-6)
Supplement: Supplementary file 1 — Supplementary Material 1 (PDF 772 KB) [file 114_2026_2083_MOESM1_ESM.pdf]

SUPPLEMENTARY MATERIAL FOR

Thermal patterns in stingless bee colonies

Charles Fernando dos Santos[1], Kedar Devkota[2], Betina Blochtein[3], Eduardo A. B. Almeida[4]

[1] Laboratório de Abelhas e Polinização, Departamento de Fitossanidade, Faculdade de Agronomia, Universidade Federal do Rio Grande do Sul, Porto Alegre, Brazil.
[2] Faculty of Agriculture, Agricultural and Forestry University, Chitwan, Nepal.
[3] Mais Abelhas Consultoria Ambiental Co., Porto Alegre, Brazil.
[4] Laboratório de Biologia Comparada e Abelhas, Departamento de Biologia, Faculdade de Filosofia, Ciências e Letras de Ribeirão Preto, Universidade de São Paulo, 14040-901, Ribeirão Preto, São Paulo, Brazil

Genus	Specie	Cerumen	Comb	Temperature
Leurotrigona	*Leurotrigona muelleri*	No	Clusters	16.5
Frieseomelitta	*Frieseomelitta varia*	No	Clusters	19.0
Leurotrigona	*Leurotrigona muelleri*	No	Clusters	19.0
Austroplebeia	*Austroplebeia essingtoni*	No	Clusters	20.0
Plebeia	*Plebeia droryana*	Yes	Brood comb	20.0
Melipona	*Melipona beecheii*	Yes	Brood comb	23.0
Melipona	*Melipona fuliginosa*	Yes	Brood comb	23.0
Partamona	*Partamona cupira*	Yes	Brood comb	23.0
Lepidotrigona	*Lepidotrigona ventralis*	Yes	Brood comb	24.0
Tetragonula	*Tetragonula carbonaria*	Yes	Brood comb	24.0
Heterotrigona	*Heterotrigona itama*	Yes	Brood comb	24.1
Tetragonisca	*Tetragonisca angustula*	Yes	Brood comb	24.3
Scaptotrigona	*Scaptotrigona depilis*	Yes	Brood comb	24.7
Tetragonula	*Tetragonula hockingsi*	No	Clusters	25.0
Frieseomelitta	*Frieseomelitta nigra*	No	Clusters	25.0
Austroplebeia	*Austroplebeia australis*	Yes	Clusters	25.0
Melipona	*Melipona colimana*	Yes	Brood comb	25.0
Melipona	*Melipona quadrifasciata*	Yes	Brood comb	25.0
Scaptotrigona	*Scaptotrigona depilis*	Yes	Brood comb	25.0
Scaptotrigona	*Scaptotrigona hellwegeri*	Yes	Brood comb	25.0
Melipona	*Melipona scutellaris*	Yes	Brood comb	25.2
Melipona	*Melipona beecheii*	Yes	Brood comb	25.4
Tetragonisca	*Tetragonisca angustula*	Yes	Brood comb	25.4
Melipona	*Melipona subnitida*	Yes	Brood comb	25.9
Tetragonula	*Tetragonula laeviceps*	No	Clusters	26.0
Melipona	*Melipona fasciculata*	Yes	Brood comb	26.0
Melipona	*Melipona scutellaris*	Yes	Brood comb	26.0
Tetragonisca	*Tetragonisca fiebrigi*	Yes	Brood comb	26.0
Melipona	*Melipona subnitida*	Yes	Brood comb	27.0
Tetragonisca	*Tetragonisca fiebrigi*	Yes	Brood comb	27.0
Austroplebeia	*Austroplebeia essingtoni*	No	Clusters	27.5
Tetragonisca	*Tetragonisca angustula*	Yes	Brood comb	27.5
Scaptotrigona	*Scaptotrigona depilis*	Yes	Brood comb	27.6
Melipona	*Melipona eburnea*	Yes	Brood comb	27.8
Melipona	*Melipona quadrifasciata*	Yes	Brood comb	27.9
Leurotrigona	*Leurotrigona muelleri*	No	Clusters	28.0
Austroplebeia	*Austroplebeia australis*	Yes	Clusters	28.0
Melipona	*Melipona bicolor*	Yes	Brood comb	28.0
Tetragonisca	*Tetragonisca fiebrigi*	Yes	Brood comb	28.1
Tetragonisca	*Tetragonisca angustula*	Yes	Brood comb	28.5
Tetragonisca	*Tetragonisca angustula*	Yes	Brood comb	28.6
Melipona	*Melipona scutellaris*	Yes	Brood comb	28.7
Melipona	*Melipona marginata*	Yes	Brood comb	28.8
Melipona	*Melipona quadrifasciata*	Yes	Brood comb	28.8
Frieseomelitta	*Frieseomelitta varia*	No	Clusters	29.0

Lepidotrigona	*Lepidotrigona ventralis*	Yes	Brood comb	29.0
Tetragonisca	*Tetragonisca weyrauchi*	Yes	Brood comb	29.0
Tetragonula	*Tetragonula carbonaria*	Yes	Brood comb	29.0
Leurotrigona	*Leurotrigona muelleri*	No	Clusters	29.2
Plebeia	*Plebeia droryana*	Yes	Brood comb	29.5
Scaptotrigona	*Scaptotrigona postica*	Yes	Brood comb	29.5
Tetragonisca	*Tetragonisca fiebrigi*	Yes	Brood comb	29.5
Plebeina	*Plebeina armata*	Yes	Brood comb	29.6
Heterotrigona	*Heterotrigona itama*	Yes	Brood comb	29.6
Melipona	*Melipona fasciculata*	Yes	Brood comb	29.6
Scaptotrigona	*Scaptotrigona depilis*	Yes	Brood comb	29.6
Plebeia	*Plebeia droryana*	Yes	Brood comb	29.7
Melipona	*Melipona interrupta*	Yes	Brood comb	29.8
Frieseomelitta	*Frieseomelitta nigra*	No	Clusters	30.0
Melipona	*Melipona beecheii*	Yes	Brood comb	30.0
Melipona	*Melipona colimana*	Yes	Brood comb	30.0
Melipona	*Melipona fuliginosa*	Yes	Brood comb	30.0
Melipona	*Melipona quadrifasciata*	Yes	Brood comb	30.0
Partamona	*Partamona cupira*	Yes	Brood comb	30.0
Scaptotrigona	*Scaptotrigona depilis*	Yes	Brood comb	30.0
Scaptotrigona	*Scaptotrigona postica*	Yes	Brood comb	30.0
Tetragonisca	*Tetragonisca fiebrigi*	Yes	Brood comb	30.0
Melipona	*Melipona compressipes*	Yes	Brood comb	30.1
Tetragonisca	*Tetragonisca angustula*	Yes	Brood comb	30.3
Scaptotrigona	*Scaptotrigona depilis*	Yes	Brood comb	30.5
Melipona	*Melipona scutellaris*	Yes	Brood comb	30.7
Lepidotrigona	*Lepidotrigona ventralis*	Yes	Brood comb	31.0
Melipona	*Melipona quadrifasciata*	Yes	Brood comb	31.0
Melipona	*Melipona rufiventris*	Yes	Brood comb	31.0
Melipona	*Melipona seminigra*	Yes	Brood comb	31.0
Melipona	*Melipona seminigra*	Yes	Brood comb	31.0
Scaptotrigona	*Scaptotrigona bipunctata*	Yes	Brood comb	31.0
Tetragonisca	*Tetragonisca fiebrigi*	Yes	Brood comb	31.1
Melipona	*Melipona fasciculata*	Yes	Brood comb	31.4
Melipona	*Melipona flavolineata*	Yes	Brood comb	31.4
Tetragonisca	*Tetragonisca angustula*	Yes	Brood comb	31.5
Tetragonisca	*Tetragonisca angustula*	Yes	Brood comb	31.6
Melipona	*Melipona rufiventris*	Yes	Brood comb	31.8
Melipona	*Melipona subnitida*	Yes	Brood comb	31.8
Scaptotrigona	*Scaptotrigona depilis*	Yes	Brood comb	31.8
Melipona	*Melipona marginata*	Yes	Brood comb	31.9
Melipona	*Melipona quadrifasciata*	Yes	Brood comb	31.9
Melipona	*Melipona rufiventris*	Yes	Brood comb	31.9
Melipona	*Melipona seminigra*	Yes	Brood comb	31.9
Plebeina	*Plebeina armata*	Yes	Brood comb	32.0
Lepidotrigona	*Lepidotrigona ventralis*	Yes	Brood comb	32.0
Melipona	*Melipona rufiventris*	Yes	Brood comb	32.0
Melipona	*Melipona rufiventris*	Yes	Brood comb	32.0
Melipona	*Melipona scutellaris*	Yes	Brood comb	32.0
Melipona	*Melipona seminigra*	Yes	Brood comb	32.0
Scaptotrigona	*Scaptotrigona depilis*	Yes	Brood comb	32.0
Scaptotrigona	*Scaptotrigona postica*	Yes	Brood comb	32.0
Tetragonisca	*Tetragonisca fiebrigi*	Yes	Brood comb	32.0
Melipona	*Melipona quadrifasciata*	Yes	Brood comb	32.2
Heterotrigona	*Heterotrigona itama*	Yes	Brood comb	32.3
Melipona	*Melipona seminigra*	Yes	Brood comb	32.3
Scaptotrigona	*Scaptotrigona depilis*	Yes	Brood comb	32.3
Melipona	*Melipona subnitida*	Yes	Brood comb	33.0
Scaptotrigona	*Scaptotrigona hellwegeri*	Yes	Brood comb	33.0
Melipona	*Melipona fasciculata*	Yes	Brood comb	33.5
Melipona	*Melipona beecheii*	Yes	Brood comb	34.0
Melipona	*Melipona scutellaris*	Yes	Brood comb	34.0

Melipona	*Melipona seminigra*	Yes	Brood comb	34.0
Scaptotrigona	*Scaptotrigona postica*	Yes	Brood comb	34.0
Trigona	*Trigona spinipes*	Yes	Brood comb	34.5
Scaptotrigona	*Scaptotrigona depilis*	Yes	Brood comb	35.0
Scaptotrigona	*Scaptotrigona postica*	Yes	Brood comb	35.0
Tetragonisca	*Tetragonisca weyrauchi*	Yes	Brood comb	35.0

References Surveyed

1. Anuar, N. H. K. *et al.* IoT platform for precision stingless bee farming. in *IEEE International Conference on Automatic Control and Intelligent Systems* 225–229 (IEEE, 2019). doi:10.1109/I2CACIS.2019.8825089

2. Sung, I. & Hozumi, S. Thermal characteristics of nests of the Taiwanese stingless bee Trigona ventralis hoozana (Hymenoptera: Apidae). *Zool. Stud.* **47**, 417–428 (2008).

3. Ali, M. A. A. C. *et al.* A review on the stingless beehive conditions and parameters monitoring using IoT and machine learning. *J. Phys. Conf. Ser.* **2107**, 1–9 (2021).

4. Macías-Macías, J. O. *et al.* Comparative temperature tolerance in stingless bee species from tropical highlands and lowlands of Mexico and implications for their conservation (Hymenoptera: Apidae: Meliponini). *Apidologie* **42**, 679–689 (2011).

5. Velthuis, H. H. W., Koedam, D. & Imperatriz–Fonseca, V. L. Temperature and brood cell production in a stingless bee The rate of brood cell production in the stingless bee Melipona bicolor fluctuates with nest box temperature. *Rev. Etol.* **2**, 141–145 (1999).

6. Cruz, D. S. *et al.* Termorregulação em abelha-sem-ferrão,. in *IV jornada Científica. Embrapa Meio-Norte* 56 (2018).

7. Correia, F. C. da S., Peruquetti, R. C. & Ferreira, M. G. Termorregulação em colônias de Melipona eburnea (Apidae: Meliponina) criadas racionalmente em Rio Branco, Acre. *EntomoBrasilis* **10**, 112–117 (2017).

8. Nieh, J. C. & Sánchez, D. Effect of food quality, distance and height on thoracic temperature in the stingless bee Melipona panamica. *J. Exp. Biol.* **208**, 3933–3943 (2005).

9. Roubik, D. W. & Peralta, F. J. A. Thermodunamics in nests of two melipona species in Brasil. *Acta Amaz.* **13**, 453–466 (1983).

10. Roldão-Sbordoni, Y. S., Gomes, G., Mateus, S. & Nascimento, F. S. Scientific note: Warming nurses, a new worker role recorded for the first time in stingless bees. *J. Econ. Entomol.* **112**, 1485–1488 (2019).

11. Oliveira, F. F., Richers, B. T. T., da Silva, J. R., Farias, R. C. & Matos, T. A. L. *Guia ilustrado das abelhas "sem-ferrão " das Reservas Amaná e Mamirauá, Amazonas, Brasil*. (IDSM, 2013).

12. Ferreira, N. da S. Temperatura colonial e tolerância térmica de melipona subnitida, uma espécie de abelha sem ferrão (Hymenoptera, Apidae, Meliponini), da caatinga. *Dissertação* **s/n**, (Universidade Federal Rural do Semi-Árido, 2014).

13. Dantas, M. R. T. Termogênese e distribuição de calor pela cria de abelhas sem ferrão e sua relação com o estágio de desenvolvimento em ambiente semiárido. (Universidade Federal Rural do Semi-Árido, 2014).

14. Souza-Junior, J. B. F. *et al.* Increasing thermal stress with flight distance in stingless bees (Melipona subnitida) in the Brazilian tropical dry forest: Implications for constraint on foraging range. *J. Insect Physiol.* **123**, 104056 (2020).

15. Jones, J. C. & Oldroyd, B. P. Nest thermoregulation in social insects. in *Advances in Insect*

Physiology **33**, 153–191 (2006).

16. Fletcher, D. J. C. & Crewe, R. M. Nest structure and thermoregulation in the stingless bee Trigona (Plebeina) denoti Vachal. *J. Entomol. Soc. South. Afr.* **44**, 183–191 (1981).

17. Vollet-Neto, A. Biologia térmica de Scaptotrigona depilis (Apidae, Meliponini): adaptações para lidar com altas temperaturas. (Universidade de São Paulo, 2011).

18. Vollet-Neto, A., Menezes, C. & Imperatriz-Fonseca, V. L. Behavioural and developmental responses of a stingless bee (Scaptotrigona depilis) to nest overheating. *Apidologie* **46**, 455–464 (2014).

19. Engels, W., Rosenkranz, P. & Engels, E. Thermoregulation in the nest of the Neotropical stingless bee Scaptotrigona postica and a hypothesis on the evolution of temperature homeostasis in highly eusocial bees. *Stud. Neotrop. Fauna Environ.* **30**, 193–205 (1995).

20. Mesquita, A. R. de, Salmento, L. L. de S., Sales Junior, O. R. de, Santos, J. H. C. dos & Araújo, F. M. A. de. Beefresh: Ferramenta de monitoramento de temperatura com Iot para colmeias de abelhas melíponas. *Brazilian J. Dev.* **6**, 52724–52740 (2020).

21. Torres, A., Hoffmann, W. & Lamprecht, I. Thermal investigations of a nest of the stingless bee Tetragonisca angustula Illiger in Colombia. *Thermochim. Acta* **458**, 118–123 (2007).

22. Amano, K., Nemoto, T. & Heard, T. A. What are stingless bees, and why and how to use them as crop pollinators? A review. *Japan Agric. Res. Q.* **34**, 183–190 (2000).

23. Jongjitvimol, T. & Wattanachaiyingcharoen, W. Distribution, nesting sites and nest structures of the stingless bee species, Trigona collina Smith, 1857 (Apidae, Meliponinae) in Thailand. *Nat. Hist. Chulalongkorn Univ.* **7**, 25–34 (2007).

24. Luttrell, B. What value a good hollow log to stingless bee colonies ?

25. Damara, I. M. G. W., Watiniasih, N. L. & Suartini, N. M. Variation of entrances, food storage and brood cells of Trigona laeviceps bees from various habitat. *Adv. Trop. Biodivers. Environ. Sci.* **1**, 50 (2018).

26. Buchwald, R., Breed, M. D. & Greenberg, A. R. The thermal properties of beeswaxes: Unexpected findings. *J. Exp. Biol.* **211**, 121–127 (2008).

27. Ramli, A. S. *et al.* A new cooling technique for stingless bees hive. in *MATEC Web of Conferences* **131**, 1–6 (2017).

28. Dantas, M. R. T. Thermogenesis in stingless bees: An approach with emphasis on brood's thermal contribution. *J. Anim. Behav. Biometeorol.* **4**, 101–108 (2016).

29. Perez, R. & Aron, S. Adaptations to thermal stress in social insects: recent advances and future directions. *Biol. Rev.* **95**, 1535–1553 (2020).

30. Abou-Shaara, H. F., Owayss, A. A., Ibrahim, Y. Y. & Basuny, N. K. A review of impacts of temperature and relative humidity on various activities of honey bees. *Insectes Soc.* **64**, 455–463 (2017).

31. Stabentheiner, A., Kovac, H., Mandl, M. & Käfer, H. Coping with the cold and fighting the heat: thermal homeostasis of a superorganism, the honeybee colony. *J. Comp. Physiol. A Neuroethol. Sensory, Neural, Behav. Physiol.* **207**, 337–351 (2021).

32. Halcroft, M. T., Haigh, A. M., Holmes, S. P. & Spooner-Hart, R. N. The thermal environment of nests of the Australian stingless bee, Austroplebeia australis. *Insectes Soc.* **60**, 497–506 (2013).

33. Ayton, S., Tomlinson, S., Phillips, R. D., Dixon, K. W. & Withers, P. C. Phenophysiological variation of a bee that regulates hive humidity, but not hive temperature. *J. Exp. Biol.* **219**, 1552–1562 (2016).

34. Becker, T. Influência da temperatura no desempenho reprodutivo de abelhas Melipona inter- rupta Latreille, 1811 (Hymenoptera: Meliponini) em condições de laboratório. *Tese de Doutorado* (Instituto Nacional de Pesquisas da Amazônia, 2019).

35. Shanks, J. L. Tetragonula carbonaria and disease: Behavioural and antimicrobial defences used by colonies to limit brood pathogens. *Doctor of Philosophy* (University of Western Sydney, 2015).

36. Proni, E. A. & Hebling, M. J. A. Thermoregulation and respiratory metabolism in two Brazilian stingless bee subspecies of different climatic distribution, Tetragonisca angustula fiebrigi and T a angustula (Hymenoptera: Apidae: Meliponinae). *Entomol. Gen.* **20**, 281–289 (1996).

37. Correia, F. C. da S., Cordeiro, M. B., Carvalho, Y. K. de & Peruquetti, R. C. Conforto térmico em colônias de Tetragonisca weyrauchi no município de Rio Branco - Acre. *Arq. Ciências Veterinárias e Zool. da UNIPAR* **18**, 237–240 (2016).

38. Silva, E. G. da *et al. MelgueiraApp: caixa de meliponicultura automatizada e aplicativo para gestão de produção de mel*. (2021). doi:10.5753/latinoware.2021.19928

39. de Castro, J. S., Cavalcante, A. de M. B., de Castro, V. J. & Silva, E. M. da. Adaptive response of Melipona subnitida ducke and colonial thermoregulation in different thermal conditions in the context to climate change. *Rev. Bras. Meteorol.* **34**, 379–387 (2019).

40. Souza Jr., J. B. F. Termorregulação e atividade de forrageamento de Melipona subnitida no bioma Caatinga. *Tese de Doutorado* (Universidade Federal Rural do Semi-Árido, 2019).

41. Loli, D. Termorregulação colonial e energética individual em abelhas sem ferrão Melipona quadrifasciata Lepeletier (Hymenoptera, Apidae, Meliponini). *Tese de Doutorado* (Universidade de São Paulo, 2008).

42. Silva, M. da C. Termorregulação e produção as Melipona scutellaris em colméias construídas com diferentes tipos de madeira. (Universidade Federal de Campina Grande, 2019).

43. Roldão, Y. S. Termorregulação colonial e a influência da temperatura no desenvolvimento da cria em abelhas sem ferrão, Melipona scutellaris (Hymenoptera, Apidae, Meliponini). (Universidade de São Paulo, 2011).

44. Becker, T. Desenvolvimento de colmeias de abelhas Melipona interrupta Latreille, 1811 (Hymenoptera: Meliponini). *Dissertação (Mestrado)* (Universidade de São Paulo, 2014).

45. Santos Neto, A. R. dos *et al.* Monitoramento da temperatura interna de colmeias de abelhas africanizadas Apis mellífera L. em Cocal – PI. *Res. Soc. Dev.* **11**, e158111234321 (2022).

46. Alburaki, M. & Corona, M. Polyurethane honey bee hives provide better winter insulation than wooden hives. *J. Apic. Res.* **61**, 190–196 (2021).

47. Roldão, Y. S. & Nascimento, F. S. Investigações sobre a termorregulação em ninhos de abelhas sem ferrão Melipona compressipes (Hymenoptera, Apidae, Meliponini). 1 (2012).

48. Moo-Valle, H., Quezada-Euán, J. J. G., Navarro, J. & Rodriguez-Carvajal, L. A. Patterns of intranidal temperature fluctuation for Melipona beecheii colonies in natural nesting cavities. *J. Apic. Res.* **39**, 3–7 (2000).

49. R Core Team. *R: A language and environment for statistical computing*. (The R Foundation for Statistical Computing, 2021).

50. Ihaka, R. & Gentleman, R. R: a language for data analysis and graphics. *J. Comput. Graph. Stat.* **5**, 299–314 (1996).

PHYLOGENETIC COMPARATIVE METHODS OF NEST TEMPERATURE

R Code

```
library(ape)

bee.tree <- read.tree("131tips_ResolveDuppl.tre", text =
"(((((Heterotrigona_itama:0.0,'Heterotrigona_itama.2':0.0,'Heterotrigona_itama.3':0.0):22.0,('Lepidotrigona_
ventralis.4':0.0,Lepidotrigona_ventralis:0.0,'Lepidotrigona_ventralis.2':0.0,'Lepidotrigona_ventralis.3':0.0):2
2.0):27.0,((('Tetragonula_carbonaria.2':0.0,Tetragonula_carbonaria:0.0):6.0,Tetragonula_hockingsi:6.0):7.
0,Tetragonula_laeviceps:13.0):36.0):24.0,((Plebeina_armata:0.0,'Plebeina_armata.2':0.0):61.0,((Austrople
beia_australis:0.0,'Austroplebeia_australis.2':0.0):2.0,(Austroplebeia_essingtoni:0.0,'Austroplebeia_essingt
oni.2':0.0):2.0):59.0):12.0):8.0,((Leurotrigona_muelleri:0.0,'Leurotrigona_muelleri.2':0.0,'Leurotrigona_muel
leri.3':0.0,'Leurotrigona_muelleri.4':0.0):71.0,((((((((Melipona_colimana:0.0,'Melipona_colimana.2':0.0):5.0,(
Melipona_eburnea:4.0,(Melipona_seminigra:0.0,'Melipona_seminigra.2':0.0,'Melipona_seminigra.3':0.0,'M
elipona_seminigra.4':0.0,'Melipona_seminigra.5':0.0,'Melipona_seminigra.6':0.0):4.0):1.0):1.0,(((Melipona_
rufiventris:0.0,'Melipona_rufiventris.5':0.0,'Melipona_rufiventris.2':0.0,'Melipona_rufiventris.3':0.0,'Melipona
_rufiventris.4':0.0):3.0,Melipona_flavolineata:3.0):0.5,(Melipona_scutellaris:0.0,'Melipona_scutellaris.2':0.0,'
Melipona_scutellaris.3':0.0,'Melipona_scutellaris.4':0.0,'Melipona_scutellaris.5':0.0,'Melipona_scutellaris.6':
0.0):3.5):2.5):1.5,(Melipona_fuliginosa:0.0,'Melipona_fuliginosa.2':0.0):7.5):10.5,(Melipona_bicolor:7.0,(Mel
ipona_marginata:0.0,'Melipona_marginata.2':0.0):7.0):11.0):4.0,(((Melipona_interrupta:7.0,Melipona_comp
ressipes:7.0):3.0,('Melipona_fasciculata.2':0.0,'Melipona_fasciculata.3':0.0,'Melipona_fasciculata.4':0.0,Mel
ipona_fasciculata:0.0):10.0):2.0,('Melipona_beecheii.3':0.0,'Melipona_beecheii.4':0.0,Melipona_beecheii:0.
0,'Melipona_beecheii.2':0.0):12.0):10.0):2.0,(('Melipona_subnitida.2':0.0,Melipona_subnitida:0.0,'Melipona
_subnitida.3':0.0,'Melipona_subnitida.4':0.0):3.0,('Melipona_quadrifasciata.5':0.0,'Melipona_quadrifasciata.
6':0.0,'Melipona_quadrifasciata.7':0.0,Melipona_quadrifasciata:0.0,'Melipona_quadrifasciata.2':0.0,'Melipon
a_quadrifasciata.3':0.0,'Melipona_quadrifasciata.4':0.0):3.0):21.0):33.0,((Partamona_cupira:0.0,'Partamon
a_cupira.2':0.0):43.0,((Trigona_spinipes:35.0,(((Scaptotrigona_postica:0.0,'Scaptotrigona_postica.2':0.0,'S
captotrigona_postica.3':0.0,'Scaptotrigona_postica.4':0.0,'Scaptotrigona_postica.5':0.0):5.4,((Scaptotrigona
_hellwegeri:0.0,'Scaptotrigona_hellwegeri.2':0.0):5.3,('Scaptotrigona_depilis.7':0.0,'Scaptotrigona_depilis.8'
:0.0,'Scaptotrigona_depilis.9':0.0,'Scaptotrigona_depilis.10':0.0,'Scaptotrigona_depilis.4':0.0,'Scaptotrigona
_depilis.5':0.0,'Scaptotrigona_depilis.6':0.0,Scaptotrigona_depilis:0.0,'Scaptotrigona_depilis.2':0.0,'Scaptotr
igona_depilis.3':0.0):5.3):0.1):0.1,Scaptotrigona_bipunctata:5.5):29.5):3.0,(('Plebeia_droryana.3':0.0,Plebei
a_droryana:0.0,'Plebeia_droryana.2':0.0):34.0,(((('Frieseomelitta_varia.2':0.0,Frieseomelitta_varia:0.0):5.0,(
Frieseomelitta_nigra:0.0,'Frieseomelitta_nigra.2':0.0):5.0):21.0,((Tetragonisca_weyrauchi:0.0,'Tetragonisca
_weyrauchi.2':0.0):17.0,((Tetragonisca_angustula:0.0,'Tetragonisca_angustula.2':0.0,'Tetragonisca_angust
ula.3':0.0,'Tetragonisca_angustula.8':0.0,'Tetragonisca_angustula.4':0.0,'Tetragonisca_angustula.5':0.0,'Te
tragonisca_angustula.6':0.0,'Tetragonisca_angustula.7':0.0):3.0,('Tetragonisca_fiebrigi.2':0.0,'Tetragonisca
_fiebrigi.7':0.0,Tetragonisca_fiebrigi:0.0,'Tetragonisca_fiebrigi.3':0.0,'Tetragonisca_fiebrigi.4':0.0,'Tetragoni
sca_fiebrigi.5':0.0,'Tetragonisca_fiebrigi.6':0.0):3.0):14.0):9.0):8.0):4.0):5.0):14.0):14.0):10.0);")

Tips <- c(
  "Heterotrigona_itama",
  "Heterotrigona_itama.2",
  "Heterotrigona_itama.3",
  "Lepidotrigona_ventralis",
  "Lepidotrigona_ventralis.2",
  "Lepidotrigona_ventralis.3",
  "Lepidotrigona_ventralis.4",
  "Tetragonula_carbonaria",
  "Tetragonula_carbonaria.2",
  "Tetragonula_hockingsi",
  "Tetragonula_laeviceps",
  "Plebeina_armata",
  "Plebeina_armata.2",
  "Austroplebeia_australis",
  "Austroplebeia_australis.2",
  "Austroplebeia_essingtoni",
```

"Austroplebeia_essingtoni.2",
"Leurotrigona_muelleri",
"Leurotrigona_muelleri.2",
"Leurotrigona_muelleri.3",
"Leurotrigona_muelleri.4",
"Melipona_colimana",
"Melipona_colimana.2",
"Melipona_eburnea",
"Melipona_seminigra",
"Melipona_seminigra.2",
"Melipona_seminigra.3",
"Melipona_seminigra.4",
"Melipona_seminigra.5",
"Melipona_seminigra.6",
"Melipona_rufiventris",
"Melipona_rufiventris.2",
"Melipona_rufiventris.3",
"Melipona_rufiventris.4",
"Melipona_rufiventris.5",
"Melipona_flavolineata",
"Melipona_scutellaris",
"Melipona_scutellaris.2",
"Melipona_scutellaris.3",
"Melipona_scutellaris.4",
"Melipona_scutellaris.5",
"Melipona_scutellaris.6",
"Melipona_fuliginosa",
"Melipona_fuliginosa.2",
"Melipona_bicolor",
"Melipona_marginata",
"Melipona_marginata.2",
"Melipona_interrupta",
"Melipona_compressipes",
"Melipona_fasciculata",
"Melipona_fasciculata.2",
"Melipona_fasciculata.3",
"Melipona_fasciculata.4",
"Melipona_beecheii",
"Melipona_beecheii.2",
"Melipona_beecheii.3",
"Melipona_beecheii.4",
"Melipona_subnitida",
"Melipona_subnitida.2",
"Melipona_subnitida.3",
"Melipona_subnitida.4",
"Melipona_quadrifasciata",
"Melipona_quadrifasciata.2",
"Melipona_quadrifasciata.3",
"Melipona_quadrifasciata.4",
"Melipona_quadrifasciata.5",
"Melipona_quadrifasciata.6",
"Melipona_quadrifasciata.7",
"Partamona_cupira",
"Partamona_cupira.2",
"Trigona_spinipes",
"Scaptotrigona_postica",
"Scaptotrigona_postica.2",
"Scaptotrigona_postica.3",

```
   "Scaptotrigona_postica.4",
   "Scaptotrigona_postica.5",
   "Scaptotrigona_hellwegeri",
   "Scaptotrigona_hellwegeri.2",
   "Scaptotrigona_depilis",
   "Scaptotrigona_depilis.2",
   "Scaptotrigona_depilis.3",
   "Scaptotrigona_depilis.4",
   "Scaptotrigona_depilis.5",
   "Scaptotrigona_depilis.6",
   "Scaptotrigona_depilis.7",
   "Scaptotrigona_depilis.8",
   "Scaptotrigona_depilis.9",
   "Scaptotrigona_depilis.10",
   "Scaptotrigona_bipunctata",
   "Plebeia_droryana",
   "Plebeia_droryana.2",
   "Plebeia_droryana.3",
   "Frieseomelitta_varia",
   "Frieseomelitta_varia.2",
   "Frieseomelitta_nigra",
   "Frieseomelitta_nigra.2",
   "Tetragonisca_weyrauchi",
   "Tetragonisca_weyrauchi.2",
   "Tetragonisca_angustula",
   "Tetragonisca_angustula.2",
   "Tetragonisca_angustula.3",
   "Tetragonisca_angustula.4",
   "Tetragonisca_angustula.5",
   "Tetragonisca_angustula.6",
   "Tetragonisca_angustula.7",
   "Tetragonisca_angustula.8",
   "Tetragonisca_fiebrigi",
   "Tetragonisca_fiebrigi.2",
   "Tetragonisca_fiebrigi.3",
   "Tetragonisca_fiebrigi.4",
   "Tetragonisca_fiebrigi.5",
   "Tetragonisca_fiebrigi.6",
   "Tetragonisca_fiebrigi.7")

names(Tips) <- bee.tree$tip.label

Thermal <- c(
24.1,
29.6,
32.3,
24.0,
29.0,
31.0,
32.0,
24.0,
29.0,
25.0,
26.0,
29.6,
32.0,
25.0,
28.0,
```

20.0,
27.5,
16.5,
19.0,
28.0,
29.2,
25.0,
30.0,
27.8,
31.0,
31.0,
31.9,
32.0,
32.3,
34.0,
31.0,
31.8,
31.9,
32.0,
32.0,
31.4,
25.2,
26.0,
28.7,
30.7,
32.0,
34.0,
23.0,
30.0,
28.0,
28.8,
31.9,
29.8,
30.1,
26.0,
29.6,
31.4,
33.5,
23.0,
25.4,
30.0,
34.0,
25.9,
27.0,
31.8,
33.0,
25.0,
27.9,
28.8,
30.0,
31.0,
31.9,
32.2,
23.0,
30.0,
34.5,
29.5,
30.0,

```
  32.0,
  34.0,
  35.0,
  25.0,
  33.0,
  24.7,
  25.0,
  27.6,
  29.6,
  30.0,
  30.5,
  31.8,
  32.0,
  32.3,
  35.0,
  31.0,
  20.0,
  29.5,
  29.7,
  19.0,
  29.0,
  25.0,
  30.0,
  29.0,
  35.0,
  24.3,
  25.4,
  27.5,
  28.5,
  28.6,
  30.3,
  31.5,
  31.6,
  26.0,
  27.0,
  28.1,
  29.5,
  30.0,
  31.1,
  32.0)

names(Thermal) <- bee.tree$tip.label

identical(bee.tree$tip.label, names(Thermal))

library(phytools)

signal.temp = phylosig(bee.tree,
                Thermal,
                method = "lambda",
                test   = TRUE,
                nsim   = 9999); signal.temp
```

EFFECT OF INVOLUCRUM (CERUMEN) ON NEST TEMPERATURE IN STINGLESS BEES

```
bee.tree <- read.tree("24species_Meliponini.tre",
                      text =
"(Leurotrigona_muelleri:71.0,(((((((Melipona_eburnea:4.0,Melipona_seminigra:4.0):2.0,(Melipona_rufiventris:3.5,Melipona_scutellaris:3.5):2.5):1.5,Melipona_fuliginosa:7.5):10.5,(Melipona_bicolor:7.0,Melipona_marginata:7.0):11.0):4.0,((Melipona_interrupta:7.0,Melipona_compressipes:7.0):3.0,Melipona_fasciculata:10.0):12.0):2.0,(Melipona_subnitida:3.0,Melipona_quadrifasciata:3.0):21.0):33.0,(Partamona_cupira:43.0,((Trigona_spinipes:35.0,((Scaptotrigona_postica:5.4,Scaptotrigona_depilis:5.4):0.1,Scaptotrigona_bipunctata:5.5):29.5):3.0,(Plebeia_droryana:34.0,((Frieseomelitta_varia:5.0,Frieseomelitta_nigra:5.0):21.0,(Tetragonisca_weyrauchi:17.0,(Tetragonisca_angustula:3.0,Tetragonisca_fiebrigi:3.0):14.0):9.0):8.0):4.0):5.0):14.0):14.0);")

plot(bee.tree,
     cex        = 0.75,
     edge.color = colpal)

summary(bee.tree)

######################
# Biological traits
######################

bee.temp <- c(
  23.2,  # Leurotrigona muelleri
  27.8,  # Melipona eburnea
  32.0,  # Melipona seminigra
  31.7,  # Melipona rufiventris
  29.4,  # Melipona scutellaris
  26.5,  # Melipona fuliginosa
  28.0,  # Melipona bicolor
  30.4,  # Melipona marginata
  29.8,  # Melipona interrupta
  30.1,  # Melipona compressipes
  30.1,  # Melipona fasciculata
  29.4,  # Melipona subnitida
  29.5,  # Melipona quadrifasciata
  26.5,  # Partamona cupira
  34.5,  # Trigona spinipes
  32.1,  # Scaptotrigona postica
  29.9,  # Scaptotrigona depilis
  31.0,  # Scaptotrigona bipunctata
  26.4,  # Plebeia droryana
  24.0,  # Frieseomelitta varia
  27.5,  # Frieseomelitta nigra
  32.0,  # Tetragonisca weyrauchi
  28.5,  # Tetragonisca angustula
  29.1   # Tetragonisca fiebrigi)

names(bee.temp) <- bee.tree$tip.label

bee.involucrum <- c(
  "No",
  "Yes",
  "Yes",
  "Yes",
  "Yes",
```

```
  "Yes",
  "Yes",
  "Yes",
  "Yes",
  "Yes",
  "Yes",
  "Yes",
  "Yes",
  "Yes",
  "Yes",
  "Yes",
  "Yes",
  "Yes",
  "Yes",
  "No",
  "No",
  "Yes",
  "Yes",
  "Yes")

names(bee.involucrum) <- bee.tree$tip.label

species <- c(
  "Leurotrigona_muelleri",
  "Melipona_eburnea",
  "Melipona_seminigra",
  "Melipona_rufiventris",
  "Melipona_scutellaris",
  "Melipona_fuliginosa",
  "Melipona_bicolor",
  "Melipona_marginata",
  "Melipona_interrupta",
  "Melipona_compressipes",
  "Melipona_fasciculata",
  "Melipona_subnitida",
  "Melipona_quadrifasciata",
  "Partamona_cupira",
  "Trigona_spinipes",
  "Scaptotrigona_postica",
  "Scaptotrigona_depilis",
  "Scaptotrigona_bipunctata",
  "Plebeia_droryana",
  "Frieseomelitta_varia",
  "Frieseomelitta_nigra",
  "Tetragonisca_weyrauchi",
  "Tetragonisca_angustula",
  "Tetragonisca_fiebrigi")

names(species) <- bee.tree$tip.label

# Phylogenetic Generalized Least Squares (PGLS)

library(caper)

ABC.temp <- data.frame(
  bee.temp = bee.temp,
  bee.involucrum = bee.involucrum,
  species = names(bee.temp))
```

```
ABC.temp$species <- as.character(ABC.temp$species)

comparative.bees <- comparative.data(
  phy = bee.tree,
  data = ABC.temp,
  names.col = "species",
  vcv = TRUE,
  warn.dropped = TRUE)

pgls.bees <- pgls(bee.temp ~ bee.involucrum,
                  lambda = "ML",
                  data   = comparative.bees); pgls.bees

summary.pgls(pgls.bees)

library(phylobase)

p4d.sp <- phylo4d(bee.tree,
                 bee.temp); head(p4d.sp); plot(p4d.sp)

names(tipData(p4d.sp))  <- sub("^dt$", "Temperature", names(tipData(p4d.sp)))
names(nodeData(p4d.sp)) <- sub("^dt$", "Temperature", names(nodeData(p4d.sp)))

library(phylosignal)

bee.lipa <- lipaMoran(p4d.sp); head(bee.lipa) # Locating the signal with LIPA

png("A.png",
    width  = 6,
    height = 5,
    units  = 'in',
    res    = 400)

A.barplot <- recordPlot()

labels(p4d.sp) <- gsub("_", " ", labels(p4d.sp))

barplot.phylo4d(p4d.sp,
               bar.col = "blue",
               center  = FALSE,
               scale   = FALSE,
               tip.cex = 1.25,
               grid.col = "blue",
               grid.lty = "dotted")

mtext(" (°C)",
      side = 1,
      line = 3.5,
      cex  = 0.9,
      adj  = .595)

A.barplot <- recordPlot()

replayPlot(A.barplot)

dev.off()
```

GENERALIZED LEAST SQUARES MODELS FOR BROOD TEMPERATURE COMPARING PERIPHERY AND ENVIRONMENT EFFECTS ON BROOD COMB

Generalized Least Squares (GLS) workflow

R Code

```
# Packages
library(nlme); library(ggplot2); library(dplyr); library(tidyr);
library(emmeans)

# ==== 1) Data ====
data <- tibble::tribble(
  ~ID, ~brood, ~periphery, ~environment,
  1, 21.0, 20.5, 17.5,
  2, 31.0, 27.5, 22.7,
  3, 25.4, 21.0, 18.2,
  4, 34.0, 30.5, 36.0,
  5, 31.0, 27.1, 22.8,
  6, 32.4, 31.3, 29.9,
  7, 30.2, 26.9, 18.9,
  8, 31.4, 27.9, 34.5,
  9, 28.1, 23.1, 20.5,
  10, 31.0, 28.0, 23.0,
  11, 31.9, 29.5, 29.9,
  12, 29.5, 28.0, 20.0,
  13, 34.0, 32.0, 31.5,
  14, 32.0, 29.0, 26.0,
  15, 39.0, 39.0, 44.0,
  16, 27.5, 18.5, 14.0,
  17, 31.5, 20.5, 24.0,
  18, 29.0, 20.0, 19.0,
  19, 35.0, 35.0, 36.0,
  20, 26.0, 16.0, 15.0,
  21, 30.0, 25.0, 25.0,
  22, 28.0, 24.0, 20.0,
  23, 34.0, 37.0, 35.0,
  24, 27.0, 12.0, 10.0,
  25, 30.0, 25.0, 22.0,
  26, 25.0, 20.0, 10.0,
  27, 30.0, 30.0, 28.0,
  28, 30.0, 21.0, 15.0,
  29, 31.5, 27.5, 31.5,
  30, 33.3, 16.0,  8.2,
  31, 36.2, 27.7, 30.2,
  32, 23.7, 19.1, 15.5,
  33, 32.0, 24.8, 25.5,
  34, 30.5, 28.9, 26.5,
  35, 30.3, 25.4, 18.0
)
```

```
# ==== 2) GLS Models ====
# Base (homoscedastic)
m0 <- gls(brood ~ periphery  environment, data = data, method = "REML")

# Heteroscedasticity as a function of 'environment' and 'periphery' (power
and exponential)
m1 <- gls(brood ~ periphery  environment, data = data,
          weights = varPower(form = ~ environment), method = "REML")

m2 <- gls(brood ~ periphery  environment, data = data,
          weights = varPower(form = ~ periphery), method = "REML")

m3 <- gls(brood ~ periphery  environment, data = data,
          weights = varExp(form = ~ environment), method = "REML")

m4 <- gls(brood ~ periphery  environment, data = data,
          weights = varExp(form = ~ periphery), method = "REML")

# Variance changing with the fitted value
m5 <- gls(brood ~ periphery  environment, data = data,
          weights = varPower(form = ~ fitted(.)), method = "REML")

# Comparison by AIC
aics <- AIC(m0, m1, m2, m3, m4, m5); aics

aics[order(aics$AIC), ]

# Automatic selection of the best (lowest AIC)
list_models <- list(m0=m0, m1=m1, m2=m2, m3=m3, m4=m4, m5=m5); list_models

best_name <- rownames(aics)[which.min(aics$AIC)]; best_name

best <- list_models[[best_name]]

cat("Best model by AIC:", best_name, "\n")

# analyzing significance of the selected model
best_ml <- update(best, method = "ML")

# null model with the SAME correlation/variance structure
null_ml <- update(best_ml, . ~ 1)

# LRT: returns the global p-value
anova(null_ml, best_ml)

# ==== 3) Graphical diagnosis ====
par(mfrow = c(2,2))
plot(fitted(best), resid(best), pch=19, main="Residuals vs Fitted",
     xlab="Fitted", ylab="Residual"); abline(h=0,lty=2)
qqnorm(resid(best)); qqline(resid(best))
plot(data$environment, abs(resid(best)), pch=19,
     main="|Residual| vs Environment");
```

```
plot(data$periphery, abs(resid(best)), pch=19,
     main="|Residual| vs Periphery")
par(mfrow = c(1,1))

# Long: factor 'site' with three levels (brood, periphery, environment)
long <- data |>
  pivot_longer(cols = c(brood, periphery, environment),
               names_to = "site", values_to = "temp") |>
  mutate(ID = factor(ID),
         site = factor(site, levels = c("environment","periphery","brood")))
# optional order

# GLS with intra-ID correlation and distinct variances by 'site'
m_gls <- gls(temp ~ site, data = long,
             correlation = corCompSymm(form = ~ 1 | ID),     # same
correlation between measurements from the same ID
             weights     = varIdent(form = ~ 1 | site),      # residual
variances by level of 'site'
             method = "REML"); m_gls

# Post-hoc test (only among brood, periphery, and environment)
emm <- emmeans(m_gls, ~ site); emm

pairs(emm, adjust = "fdr")   # multiple comparisons among the 3 levels
```
